# The Study of Alanine Transaminase Activity in Tissues of Silkworm (*Bombyx mori*) via Direct Analysis in Real-Time (DART) Mass Spectrometry

**DOI:** 10.3390/molecules28104131

**Published:** 2023-05-16

**Authors:** Guohua Wu, Lei Jiang, Jianjun Guo, Wushuang Li, Lin Ma, Bozhi Tang, Charles C. Liu

**Affiliations:** 1 College of Biotechnology, Jiangsu University of Science and Technology, Zhenjiang 212100, China; lwsjy8y@163.com (W.L.); ma_lin_1988@126.com (L.M.); tang.brody@gmail.com (B.T.); 2 College of Environmental and Chemical Engineering, Jiangsu University of Science and Technology, Zhenjiang 212100, China; wckjl980102@163.com; 3 College of Agriculture, Anshun University, Anshun 561000, China; jianjunguo868@163.com; 4 ASPEC Technologies Limited, Beijing 100101, China; charles.c.liu@aspectechnologies.com

**Keywords:** ALT, DART-MS, *Bombyx mori*, biochemical analysis

## Abstract

Alanine transaminase (ALT) is an important amino acid-metabolizing enzyme in silkworm *Bombyx mori* L., and is mainly involved in transferring glutamate to alanine (serving as an essential precursor in silk protein synthesis) through transamination. Therefore, it is generally believed that silk protein synthesis in the silk gland and the cocoon quantity increase with the increase in ALT activity to a certain extent. Here, a novel analytical method was developed to determine the ALT activity in several key tissues of *Bombyx mori* L. including the posterior silk gland, midgut, fat body, middle silk gland, trachea and hemolymph, by combining the direct-analysis-in-real-time (DART) ion source with a triple-quadrupole mass spectrometer. In addition, a traditional ALT activity assay, the Reitman–Frankel method, was also used to measure ALT activity for comparison. The ALT activity results obtained via the DART-MS method are in good agreement with those obtained via the Reitman–Frankel method. However, the present DART-MS method provides a more convenient, rapid and environmentally friendly quantitative method for ALT measurement. Especially, this method can also monitor ALT activity in different tissues of *Bombyx mori* L. in real time.

## 1. Introduction

Silkworm *Bombyx mori* L. is a holometabolous lepidopteran insect and an agriculturally important species with high economic value; it has been completely domesticated by human beings for thousands of years. More recently, *Bombyx mori* L. was used as a model creature in scientific research [1,2] and other fields. The growth and development of *Bombyx mori* can be divided into four stages: eggs, larvae, pupa and adults. The fifth instar is the metamorphosis transition period from larvae to pupae and is also a stage of a large amount of silk biosynthesis and spinning. During this period, larvae ingest almost all nutrients for the whole process of their remaining life [2]. The posterior silk gland, midgut, fat body, middle silk gland, trachea and hemolymph are important tissues in the silkworm’s growth and development, and have various functions such as nutrition absorption, digestion, fibroin synthesis, and even immunity, etc.

Alanine aminotransferase (ALT) is a vital amino acid-metabolizing enzyme in silkworms, which can transform glutamate (its content is very high in mulberry leaves) to alanine through transamination under specific conditions [3,4]. The transformation is rather important since the content of alanine in mulberry leaves, i.e., the only food of silkworms, is very low. However, the content of alanine is rather high (more than 30%) in final produced silk [5]. This means that a large amount of alanine is required for silk protein synthesis, and relies mainly on the catalytic synthesis of alanine in silkworm tissues. In other words, the silkworm converts glutamate to alanine under the action of ALT, which provides raw materials for the synthesis of silk protein. ALT obviously plays a key role on the synthesis of silk protein, thus affecting the yield and quality of the silk. Therefore, establishing an accurate and rapid detection method to determine the ALT activity of silkworm is of great significance for the benefits of sericulture and related research fields.

The Reitman–Frankel method is the most widely used one due to its advantages such as the simple instrument and low cost. However, this method has the following disadvantages: the operation steps are usually tedious and laborious, the dangerous solvent 2,4-dinitrophenylhydrazine (2,4-DNPH, a chemical with flammable and explosive properties, which requires strict conditions of use and storage) is needed, and the products of the enzyme reaction are difficult to handle and easily pollute the environment. Therefore, it is of great significance to find a new, simple and environmentally friendly method to determine ALT activity.

Biochemical analysis is generally performed using biochemical assays, chromatography, fluorescence spectroscopy, immunohistochemistry, etc. In recent years, ambient mass spectrometry (AMS) has been applied in the research of biochemical analysis with increasing frequency, and has been widely used in various fields such as medicine, public health and food production, showing tremendous capabilities and distinctive contributions. AMS enables in situ, direct and rapid sample analysis without complicated sample preparation, and time savings [6,7]. In biochemical analysis, mass spectrometry is becoming an increasingly important analytical platform for metabolomics. Compared to nuclear magnetic resonance, mass spectrometry is more sensitive and allows the rapid analysis of samples, which increases the scope of applications in metabolomics [8].

Among the various AMS ionization sources, using direct-analysis-in-real-time (DART) ion sources is a non-surface-contact, thermal-analytical atmospheric pressure ionization technique that combines the simplicity and sensitivity of plasma technology in AMS, and can directly analyze solids, liquids, and gases with little to no sample preparation [9]. Since being introduced by Cody in 2005 and gradually commercialized, it has been extensively performed in diverse fields due to its high detection speed, strong anti-interference ability, high throughput, and direct analysis of complex matrix samples [10]. At present, DART is one of the most commonly used AMS methods in biochemical analysis [11,12]. For example, it was reported that the DART-MS method has been used for the analysis of dried blood spots for the detection of phenylketonuria in newborns and quantification of metabolites in human plasma [13]. DART-MS technology was also used in various biochemical analyses of the natural moisturizing factor in the stratum corneum, breast cancer, ovarian cancer, carp muscle and chicken meat metabolomics [14,15,16,17].

In addition, if a large amount of nonvolatile salts, such as phosphates, are utilized in the experiment, they not only contaminate the ion source and the mass spectrometer inlet, but also generates ion suppression and a decrease in the sensitivity of the instrument as well. Compared with traditional ionization technology, DART has strong salt tolerance [18]. Studies have shown that DART-MS is able to tolerate eluents containing a 120 mM phosphate buffer (PBS) and produces no contamination and ion suppression. This confirms the excellent salt tolerance of the DART ion source and also expands its application range to various sample types.

In this article, a novel DART-MS method, with the advantages of simplicity, rapidness, good reproducibility and high sensitivity, is developed to determine the ALT activity in different tissues of *Bombyx mori*. Moreover, the amount of alanine generated at different reaction times can be detected at any time to monitor the progress of the enzyme reaction process in real time.

## 2. Results and Discussion

### 2.1. Optimization of DART-MS/MS Experimental Parameters

#### 2.1.1. Effect of Gas Temperature in DART Ionization Source

**Figure 1:**
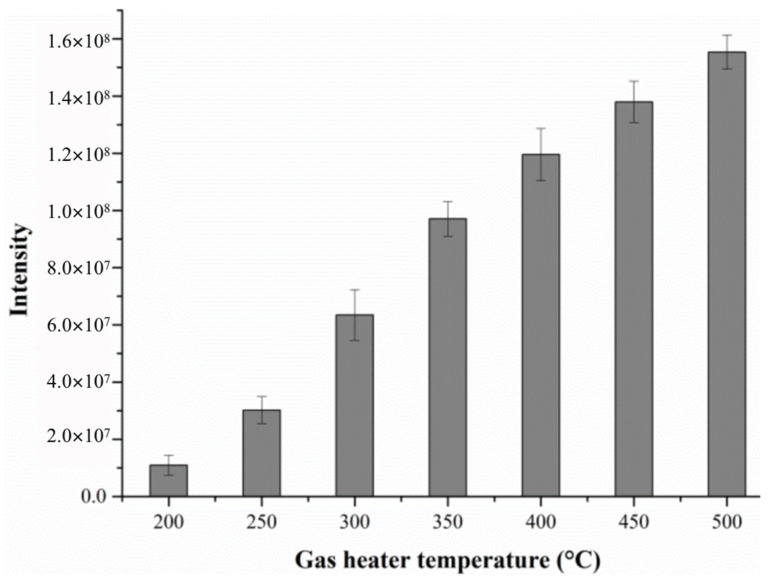
The effect of gas temperature on the response intensity of alanine.

In the present method, the temperature of the gas heater is one of the most important parameters. The heating temperature should be properly optimized to generate the maximal signal intensity, and the substrate should only generate the least amount of background ions simultaneously [19]. In this experiment, we investigated the effect of gas heater temperature on the alanine response intensity by changing the heater temperature of the DART ion source from 200 °C to 500 °C in 50 °C intervals, as shown in Figure 1. It can be observed from the figure that the response intensity of alanine increases with the increase in temperature.

**Figure 2:**
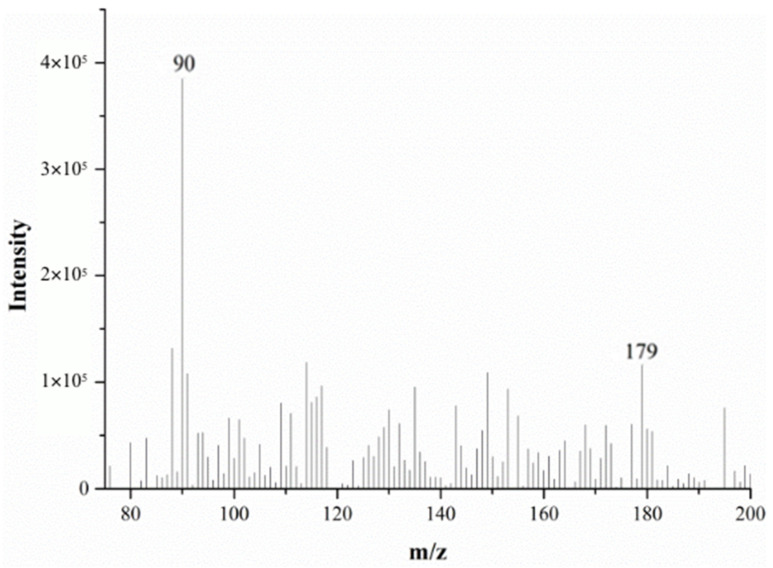
The mass spectra of alanine monitored via DART-MS at 200 °C.

**Figure 3:**
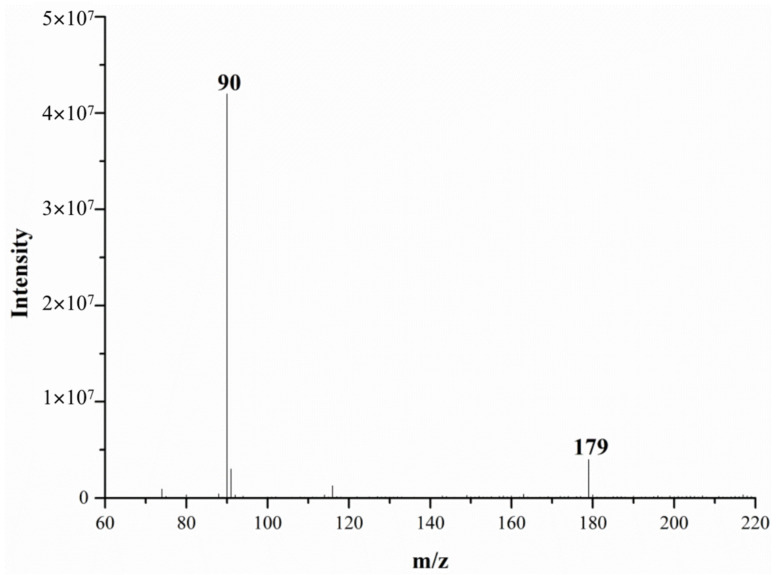
The mass spectra of alanine monitored via DART-MS at 500 °C.

Figure 2 and Figure 3 show the DART-MS mass spectra of alanine at 200 °C and 500 °C. From these figures, it can be seen that alanine can be detected via DART-MS at lower temperature (e.g., 200 °C), but the response is relatively low and the spectral noise is high.

Obviously, when the gas heater temperature gradually increases, the evaporation and desorption rate of the components in the sample is accelerated, and more analytes enter the MS detector [20]. Subsequently, more ionized target compounds are formed, and the corresponding ion intensities also progressively increases, leading to an increase in the sensitivity of detection. Therefore, 500 °C was chosen as the optimum temperature for the working gas here.

#### 2.1.2. Effect of Grid Electrode Voltage

The grid electrode is located at the exit of the DART ion source behind an insulating cap, and its voltage also seriously affects the outcome of DART-MS analysis. The grid not only serves as an ion repeller but also has other functions such as removing ions of opposite polarity, thus preventing signal loss of analyte via ion–ion recombination [21]. Borges et al. studied the effect of grid electrode voltage on the response variation of organometallic compounds and found that the grid electrode can eliminate reactive ions in the atmosphere, such as NO^+^, and can also reduce the chemical background noise [22].

**Figure 4:**
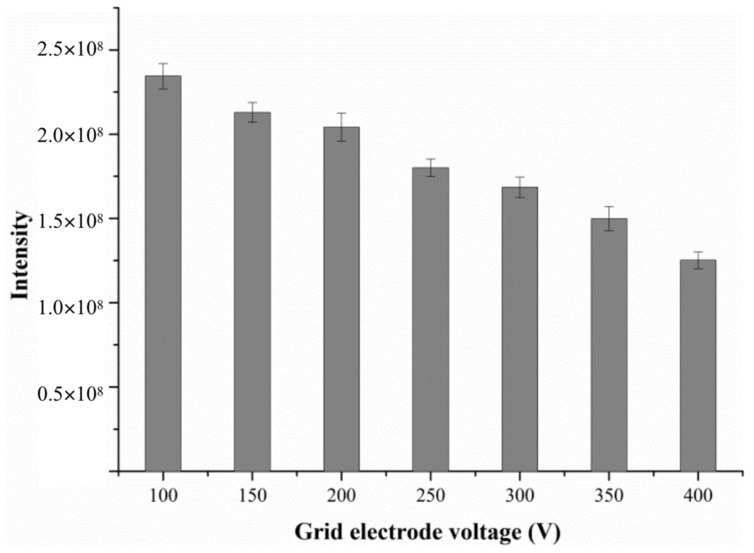
The effect of grid electrode voltage on the response intensity of alanine.

In order to obtain the optimal mass spectra and good signal intensity for target compounds, the grid electrode voltage was optimized in the pre-experiment by raising it from 100 V to 400 V, as shown in Figure 4. The data shows that as the grid electrode voltage increases from 100 V to 400 V, the response intensity of alanine gradually decreases. Thus, the grid electrode voltage was set to 100 V.

#### 2.1.3. Effect of Sample Presentation Speed

The sample was introduced into the DART ion source through a software-controlled linear rail. Typically, the linear rail speed was set between 0.2 and 10 mm/s. The sample was desorbed as it passed through the ionization region of the DART source. Thus, the speed of the sample which was pushed through the region affected the time duration of the DART desorption ionization experiment, and the MS signal intensity.

Obviously, the lower sample introduction speed was conducive to the sufficient contact between the desorption gas and sample molecules, which allowed more analytes to enter the mass spectrometer. The results demonstrated that lower-speed sampling produced not only a better signal intensity but also higher reproducibility. Similar results have also been reported by other groups [23,24]. It was found that when the sampling speed was 1 mm/s, only a few more intense signals could be detected by the mass spectrometer, which correspond to the light ions, while a high response intensity could be observed with DART-MS with a rail speed range of 0.2–0.6 mm/s.

**Figure 5:**
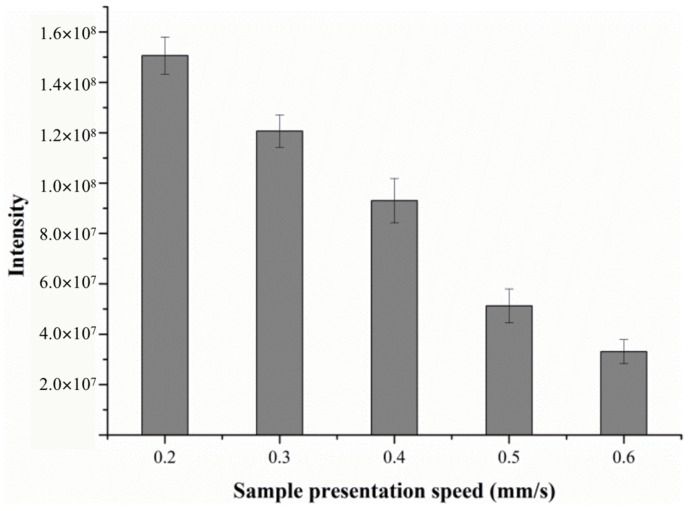
The effect of sample presentation speed on the response intensity of alanine.

To ensure high-sensitivity and better-quality mass spectra, the effects of sample presentation speed on signal intensity were tested from 0.2 mm/s to 0.6 mm/s. The response intensity of alanine under various speeds is shown in Figure 5. As can be seen, the signal intensity decreases with the increase in sampling speed. There were significant differences between signal intensity and different rail speed, so we selected the sample presentation speed of 0.2 mm/s throughout the subsequent study.

### 2.2. The Characterization of Amino Acid via DART-MS/MS

To measure the enzyme activity of ALT, a concentration of alanine is needed, so the characterization of alanine in DART-MS is necessary.

#### 2.2.1. Alanine MS in Positive Ion Mode

**Figure 6:**
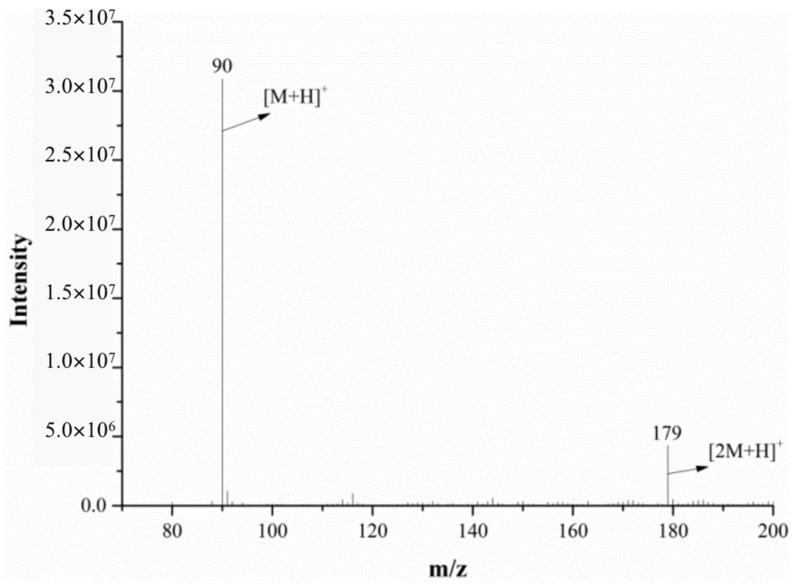
The mass spectra of alanine monitored via DART-MS in the positive ion mode.

**Figure 7:**
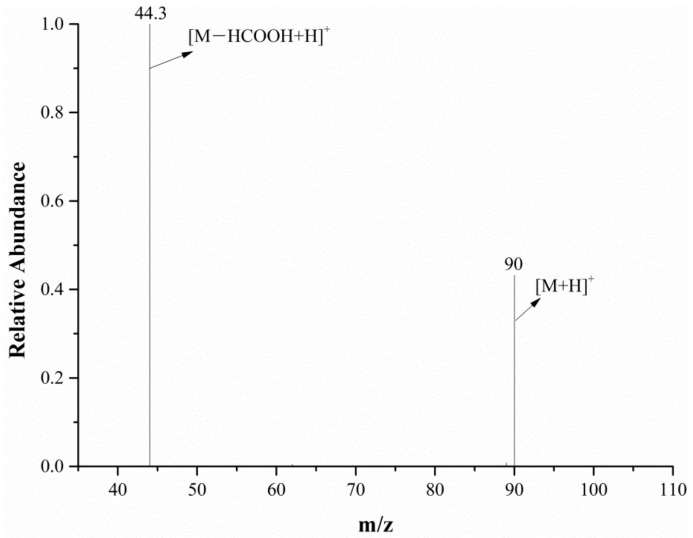
The MS/MS spectra of the *m*/*z* 90 ion from the alanine in the positive ion mode.

**Figure 8:**
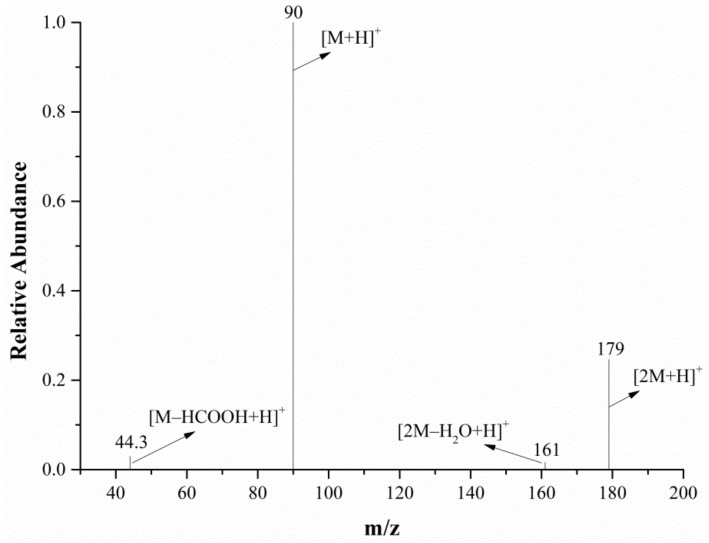
The MS/MS spectra of the *m*/*z* 179 ion from the alanine in the positive ion mode.

The full-scan mass spectrum of alanine is shown in Figure 6; both the mass spectrometer and DART ion source were in the positive ion mode. The relative molecular mass of alanine is 89.09. As can be seen from Figure 6, the characteristic alanine ions were *m*/*z* 90 and *m*/*z* 179. After the DART-MS/MS analysis of the two characteristic ions, as shown in Figure 7 and Figure 8, it was indicated that *m*/*z* 90 and *m*/*z* 179 were the [M+H]^+^ and dimeric ion [2M+H]^+^, respectively. As seen in Figure 7, *m*/*z* 44.3 was the only product ion of *m*/*z* 90, which shows that *m*/*z* 90.0 is more inclined to lose HCOOH to produce m/z 44.3 than to dehydrate. In addition to *m*/*z* 90 and *m*/*z* 44.3, the product ion *m*/*z* 179 also contained the low-intensity ion *m*/*z* 161, indicating that the dimeric ion [2M+H]^+^ underwent a dehydration reaction during dissociation and then generated *m*/*z* 161.

#### 2.2.2. Alanine MS in Negative Ion Mode

**Figure 9:**
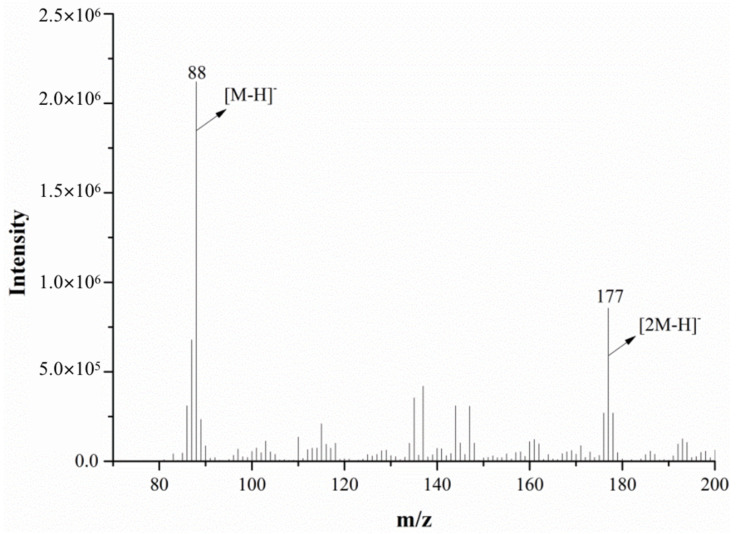
The mass spectra of alanine monitored via DART-MS in the negative ion mode.

The mass spectrum of alanine in the negative ion mode (for both the mass spectrometer and DART ion source) is shown in Figure 9. As seen in the figure, the spectrum has a low ion response intensity, a complex background and many impurity peaks. The characteristic parent ions of alanine were mainly [2M−H]^−^ *m*/*z* 88 and *m*/*z* 177. After the MS/MS analysis of *m*/*z* 177, *m*/*z* 88 was the only fragment ion obtained under the product ion’s scan mode, suggesting that *m*/*z* 177 may be a dimeric ion, [2M−H]^−^, of alanine.

By comparing the mass spectra of alanine in the positive and negative ion mode, it was found that the spectrum in the negative ion mode has a lower response intensity, a more complex background and more impurity peaks, which will affect the identification of important ion peaks in the mass spectrum; in contrast, the spectrum in the positive ion mode is better, so both the mass spectrometer and the DART ion source were set to positive ion mode in the subsequent experiment.

In the characteristic ions of alanine, *m*/*z* 90 has a higher response intensity and only produces one daughter ion, *m*/*z* 44.3, so *m*/*z* 90→*m*/*z* 44.3 was chosen as the quantitative ion pair, and subsequent analysis experiments were performed in the selected reaction monitoring (SRM) mode.

### 2.3. Determination of Alanine and ALT Activity in the Tissues of Silkworm by DART-MS

In this experiment, ALT from the tissues of silkworms acted on the substrate composed of glutamate and pyruvate, to produce alanine and ketoglutarate. The produced alanine was then determined via DART-MS, the content of which was brought into the enzyme activity formula to acquire the ALT activity in the tissue sample. During the assay, ions *m*/*z* 90 and *m*/*z* 44.3 were used as the quantitative ion pair, and the external standard method was applied.

#### 2.3.1. Calibration Curve

**Figure 10:**
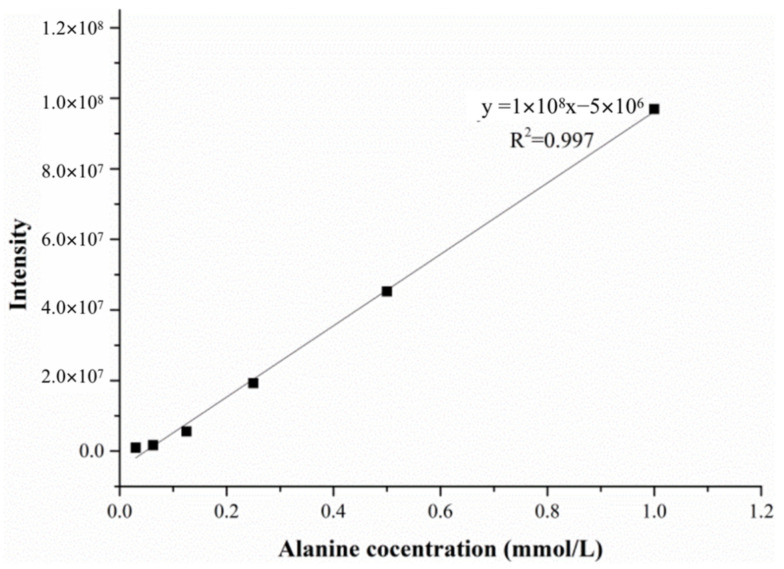
The linear calibration curve of the alanine solution.

In order to eliminate the effect of matrix effects, a matrix solution similar to the that of the enzyme reaction was added during the establishment of the calibration curve, so that the standard solution and the sample solution had similar ionization conditions. The matrix solution was composed of 0.004 mol/L sodium pyruvate, 0.067 mol/L PBS and ultrapure water at a volume ratio of 1:2:1. The calibration curve solution was prepared in a 4:1 ratio of the matrix solution and alanine standard solution, and the concentration of the alanine in the calibration curve solution ranged from 0.03 mmol/L to 1.00 mmol/L. The linear calibration curve and correlation coefficients were *y* = 1 × 10^8^*x* − 5 × 10^6^ and *R*^2^ = 0.997, as shown in Figure 10.

#### 2.3.2. Precision and Spike Recovery Tests

To evaluate the precision and accuracy of the method, the posterior silk gland was used as an example. Tissue homogenates of the posterior silk glands, with three concentrations levels, low (0.2%), medium (0.5%), and high (1%), were prepared. The precision (RSD%) of the three groups of posterior silk glands solution were 10.90%, 4.61% and 5.10%, respectively. For the spiked recovery, alanine was added to the posterior silk gland solution (0.5%) to evaluate the accuracy. To be specific, equal amounts of alanine at different concentrations (0.16 mmol/L, 0.20 mmol/L, and 0.22 mmol/L) were added to three identical reaction solutions. The spiked recovery was obtained, and was between 97.61% and 111.92% (see Table 1).

### 2.4. The Evaluation of the ALT Activity in Silkworm Tissues

The variations in ALT activity of six tissues (posterior silk gland, midgut, fat body, middle silk gland, trachea and hemolymph) from fifth-instar silkworm larvae were evaluated with the change in time, since the fifth instar usually includes seven days. Both the DART-MS method and Reitman–Frankel method were used to determine the ALT activity in these tissues for comparison. The corresponding results are as follows.

#### 2.4.1. Quantifying ALT Activity in the Hemolymph of Silkworms

**Figure 11:**
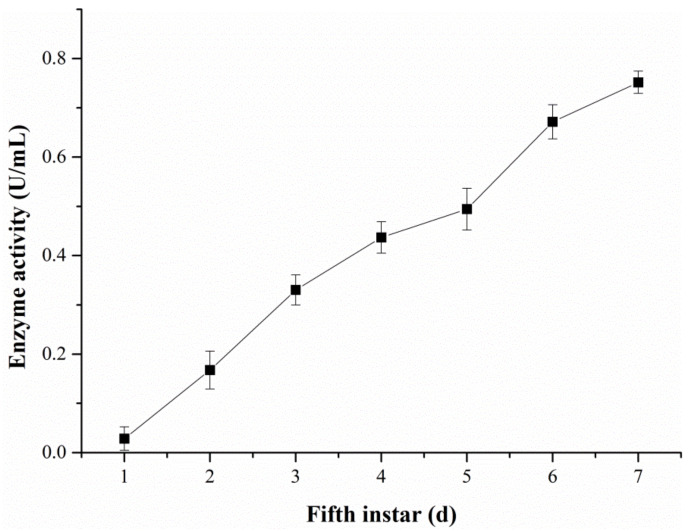
The ALT activity in the hemolymph determined via the DART-MS method.

**Figure 12:**
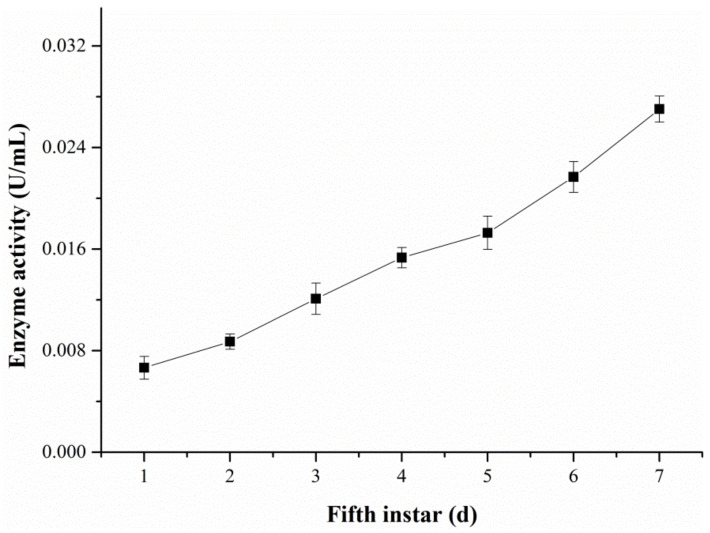
The ALT activity in the hemolymph determined via the Reitman–Frankel method.

The changes in the ALT activity in hemolymph were examined with the DART-MS and Reitman–Frankel methods, respectively (Figure 11 and Figure 12). Figure 11 reveals that the activity level of ALT detected via DART-MS increased progressively from the first day to the seventh day of the fifth-instar larvae. Although little fluctuation exists in the results, the enzyme activity determined via the Reitman–Frankel assay presented a similar upward trend in total.

#### 2.4.2. Determination of ALT Activity in Tissues of *Bombyx mori* L.: The Posterior Silk Gland, Midgut, Fat Body, Middle Silk Gland and Trachea

**Figure 13:**
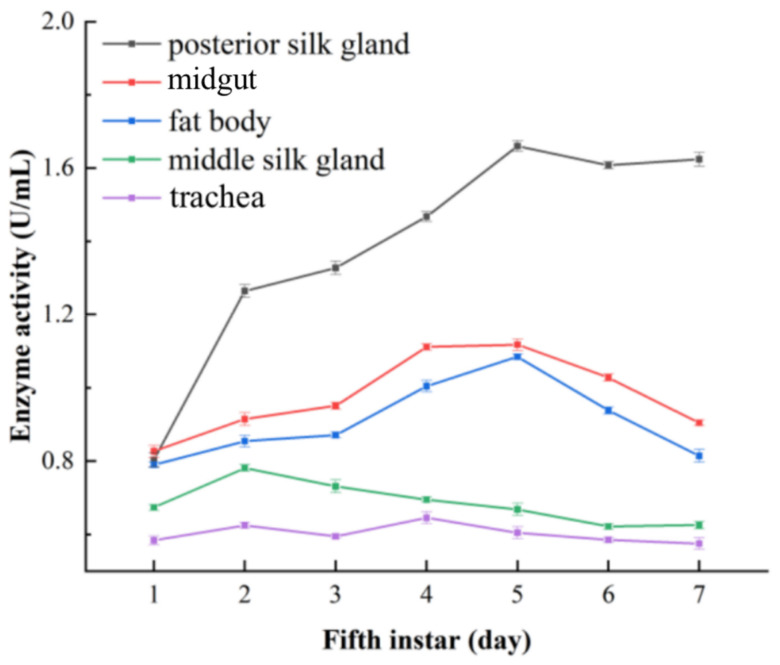
The changes in ALT activity in silkworm tissues determined via the DART-MS method.

**Figure 14:**
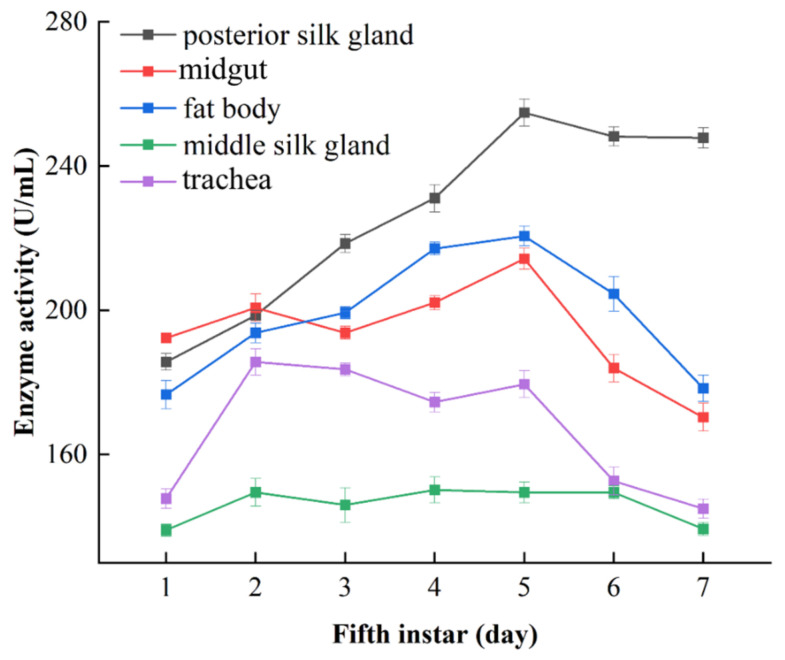
The changes in ALT activity in silkworm tissues determined via the Reitman–Frankel method.

Both the DART-MS and Reitman–Frankel methods were also used to determine the ALT activity in five other tissues (the posterior silk gland, midgut, fat body, middle silk gland and trachea) from silkworm larvae, and the results are shown in Figure 13 and Figure 14.

The ALT activity in the posterior silk gland was slightly lower at the initial stage of the fifth instar, which then sharply increased and reached the maximum on the fifth day. The variation tended to become less pronounced in the middle and late period. Figure 13 showed that ALT activity in the posterior silk gland was significantly higher than that in the other tested tissues. The ALT activity in the midgut increased gradually at the prophase, rose to its maximum on the fourth day, and declined in the following days. The ALT activity of the fat body also showed an upward trend in the start stage, reached its maximum on the fifth day and then decreased in subsequent days. The ALT activity in the middle silk gland had a high level in the early fifth instar, increased slowly in the middle stage and diminished in the late stages. The ALT activity in the trachea was the lowest in all the tested tissues and fluctuated in a small range during the seven days of the fifth instar, which might imply that the trachea is not an important tissue for ALT.

Judging from the overall level of ALT activity in the five tissues, the changing trends derived from the Reitman–Frankel method were similar to those derived from DART-MS. A few different variations emerged in the ALT activity of midgut, the content of which was higher than that of the posterior silk gland and fat body in the initial stage.

#### 2.4.3. Limit of Detection

**Figure 15:**
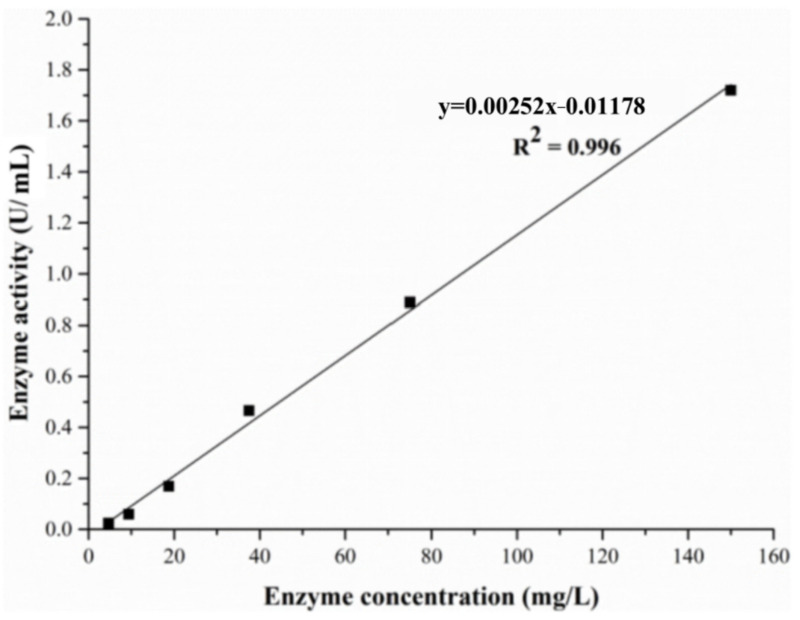
The limit of detection curve of the ALT standard solution.

The limit of detection (LOD) was determined with an ALT standard solution of various concentrations. The ALT standard solution of a concentration of 150 mg/L was diluted with PBS in a series of gradient solutions, and the concentrations were from 150 mg/L to 0.3 mg/L. The ALT activity was determined at different enzyme concentrations (Figure 15). When the concentration was in the range of 4.7–150 mg/L, the measured ALT activity and the concentration of ALT showed a good linear relationship, and the correlation coefficient *R*^2^ = 0.996, indicating that the level of the ALT activity was related to the amount of enzyme added. When the ALT concentration was lower than 4.7 mg/L, the alanine content was close to or even lower than the measured value of the control group. These results demonstrated that ALT failed to catalyze reactions when its concentration was lower than 4.7 mg/L, which means that the LOD of ALT was 4.7 mg/L in this experiment.

### 2.5. The Relation between the Variation in ALT Activity and the Production of Silk Protein

The fifth instar is the transitional period between the silkworm’s stages of being a larva and an adult, and it is also the period during which it carries out a large amount of biosynthesis of silk and spinning.

Additionally, the third day of the fifth instar, as a boundary for the development of larvae [25], is a crucial time for silkworm. From the third day on, an abundance of silk proteins are synthesized in the silkworm’s body. As an important amino acid metabolism enzyme, ALT makes a substantial contribution towards the synthesis of silkworm silk proteins. The ALT activity measured in the current study gradually decreased in the middle and late period of the fifth instar, which indicates that the ALT was consumed by larvae for the synthesis of silk protein. The normal growth and development of the posterior silk gland is a vital part for the synthesis and secretion of silk protein [26]. Thus, the ALT activity in the posterior silk glandwais at the highest level compared with those in other tissues. The results from DART-MS method are in good agreement with those acquired from the Reitman–Frankel assay, suggesting that this novel technique, i.e., the DART-MS method, is a feasible and effective alternative analysis method.

## 3. Materials and Methods

### 3.1. Chemicals and Reagents

L-glutamic acid, L-alanine, ketoglutarate and pyruvic acid sodium were purchased from Shanghai Aladdin Biochemical Technology Co., Ltd. (Shanghai, China). The alanine aminotransferase standard was obtained from Sigma–Aldrich (St. Louis, MO, USA). The alanine aminotransferase assay kit was purchased from Nanjing Jiancheng Bioengineering Institute (Nanjing, China). Ultrapure water (18.2 MΩ cm) was produced in-house by a Milli-Q system (Millipore, Milford, MA, USA).

### 3.2. Samples

#### 3.2.1. Preparation of Tissues from Silkworm Larvae

*Bombyx mori* silkworms Qingsong × Haoyue, were obtained from the Sericulture Institute of Chinese Academy of Agricultural Sciences and reared with standard rearing techniques from hatching to the fifth instar. In this experiment, 10 larvae were selected randomly for each group. The silkworm body tissues including the posterior silk gland, midgut, middle silk gland, fat body, trachea and hemolymph were sampled from the first day to the seventh day of the fifth instar. The proleg tip of *Bombyx mori* was cut to collect hemolymph, and 1 mg of an antioxidant was added to the sample tubes to prevent melanization. The hemolymph was centrifuged at 10,000 rpm for 10 min, and then the supernatant was collected. All of the samples were stored at −80 °C prior to the experiments.

#### 3.2.2. Extraction of ALT

The *Bombyx mori* tissues were accurately weighed and added into 25 mL conical flasks, and a volume of physiological saline that was nine times the weight of the tissues (for the Reitman–Frankel method) or 0.067 mol/L PBS (for the DART-MS method) was also added according to the ratio of the weight:volume = 1:9. Then, samples were homogenized mechanically in an ice water bath to obtain the 10% tissue homogenate, and were centrifuged at 2500 rpm for 10 min. The supernatant was taken and then diluted with physiological saline (or 0.067 mol/L PBS) to the appropriate concentration to be tested.

### 3.3. The Expression of ALT Activity Unit

Reaction principle:

(1) $$L-\text{Glutamic acid}+\text{Pyranic acid}\overset{ALT}{↔}L-\text{alanine}+\alpha -\text{ketoglutarate}$$

The ALT activity unit in our experiment is defined as treating 1 mL of an enzyme liquid in a water bath for a certain time at 37 °C, after which 1 μmol of alanine is generated. This amount is regarded as an enzyme activity unit. The enzyme activity is calculated as follows (unit: U/mL):

(2) $$\text{ALT activity unit}=\frac{\left(\text{Measured value}-\text{Blank value}\right)*\text{Total volume}}{\text{Enzyme volume}}$$

### 3.4. Effect of Reaction Time on the Determination of Enzyme Activity

In order to study the effect of the reaction time on the determination of enzyme activity, the products (samples) obtained after the reaction for different times (1, 2, 3, 5, 7, 10, 13, 16, 20 and 25 min) were taken immediately for DART-MS/MS analysis, i.e., measurement of the activity of the enzyme.

### 3.5. Instrumentation

#### 3.5.1. Mass Spectrometer

A TSQ Quantum Access MAX triple quadrupole mass spectrometer (Thermo Scientific, San Jose, CA, USA) was employed in the analyses. The optimized instrument conditions were as follows: the capillary temperature was 260 °C, the tube lens offset was 57 V, the skimmer offset was off, the collision pressurewas 0 mTorr and the collision energy was 0 eV. The scan mode of mass spectra was full-scan, and the mass spectra were recorded across the range *m*/*z* 50–400.

In our experiment, the MS/MS analyses were carried out in the SRM mode with a collision cell (Q2). The conditions were a a collision pressure of 1.5 mTorr; collision energy of −10 eV and the collision cell was supplied with argon gas (99.999% purity). All of the parameters listed above were optimized in the SRM mode.

The instrument was calibrated with polytyrosine dissolved in methanol in accordance with the manufacturer’s manual. All data analyses and peak integrations were performed with the Thermo Xcalibur^TM^ software suite (XCALI-97352).

#### 3.5.2. DART SVP Ionization Source

The study was realized with a DART SVP ionization source (Ion Sense, Saugus, MA, USA), which was connected to the mass spectrometer by the VAPUR interface (Ion Sense, Saugus, MA, USA). The sampling device consisted of glass sample sticks (Ion Sense, Saugus, MA, USA) and a 12 Dip-It glass-tip linear rail that passed through the gap between the DART ion source and the ceramic ion sampling tube at a constant speed, so as to introduce the samples into the mass spectrometer. The glass sample sticks had to be washed and dried firstly, then secured on an engineered block. After that, 1 μL of the samples was drawn and deposited on the bottom tips of glass sticks through a pipette.

The DART operating parameters were set as follows: the ion mode was positive, the sampling speed was 0.2 mm/s, the gas temperature was 500 °C, the gas pressure was 0.3 Mpa, and the grid electrode voltage was 100 V. The membrane pump pressure was set as 12.0 kPa. Compared with high-purity helium, high-purity nitrogen (99.999%) is cheaper, which is conducive to method development and cost saving. The key point is that experiments proved that high-purity nitrogen was able to meet the needs of our experimental analysis. Thus, high-purity nitrogen was applied as both the standby and running gas.

### 3.6. Reitman-Frankel Method

For the assay group, the matrix liquid (containing glutamate and pyruvate) was pre-warmed for 5 min at 37 °C, then 20 μL of the matrix liquid was taken into the well in 96-well plate. The liquid was mixed with 5 μL of the sample and was incubated at 37 °C for 30 min. After adding 20 μL of a 2,4-DNPH solution, the mixtures were incubated at 37 °C for another 20 min. Then, 200 μL of 0.4 mol/L NaOH liquid was added to the well to terminate the reaction. The well was kept at room temperature for 15 min, and the optical density (OD) value of each well was measured with a microplate reader at 510 nm.

For the control group, there were some differences in the order of adding reagents and the steps above. Namely, compared with the assay group, the sample here was added after 2,4-DNPH being added into the solution, and the other steps were the same as those in the assay group.

### 3.7. Preparation of Samples in This Method

For the assay group, 0.2 mL of 5 mmol/L glutamic acid, 0.2 mL of 4 mmol/L sodium pyruvate and 0.4 mL of 0.067 mol/L PBS (pH 7.4) were added to a centrifuge tube, then 0.2 mL of the ALT liquid (the supernatant of the tissue homogenate or hemolymph) was added and the tube was shaken thoroughly. The mixture was heated in a water bath at 37 °C for 10–20 min, then was put into hot water subsequently.

For the control group, the supernatant of the homogenized tissue (or hemolymph) in the assay group was replaced with 0.2 mL of ultrapure water, and the others were consistent with those in the assay group.

## 4. Conclusions

In the present work, an analytical method was developed with direct analysis in real time-mass spectrometry (DART-MS) to determine the activity of ALT, which was extracted from the tissues of the fifth-instar silkworms and has a significant influence on the synthesis of silk protein from the silkworm body. For the DART ionization, several parameters including the gas heater temperature, the grid electrode voltage and the sample presentation speed were optimized to guarantee optimum results. The experimental data demonstrated that a higher signal intensity can be obtained by measuring the analyte in the SRM mode under the positive ion mode.

According to verification analysis, the results from DART-MS are tallied with the ones from the conventional Reitman-Frankel method. As is well-known, the time used for the experiment is as critical as the results in the experimental research. This new technique not only ensures operator and environmental safety, but also achieves speed and accuracy. The DART-MS method allows the simple, direct and rapid analysis of alanine transaminase activity, especially its salt tolerance, which shows unique and excellent performance. Compared with traditional analytical approaches (such as the Reitman–Frankel method), this novel method represents an effective, operationally safe and environmentally friendly alternative, and promises to be a potential tool in biochemical applications. As an important aminotransferase in silkworm, ALT can convert glutamate to alanine, thus affecting silk fibroin synthesis. Therefore, the accurate determination of transaminase in different tissues of silkworm plays an important role in grasping the change rule of transaminase content in silkworm tissues and the impact of these changes on the physiological and biochemical processes of silkworm. It can be seen that this method for determining ALT activity established in this study has important practical significance for silkworm silk production.

## Figures and Tables

**Table 1:** The spiked recovery of the DART-MS method (average content ± SD, μmol).

Sample	Measurement (μmol)	Spiked (μmol)	Spiked Measurement (μmol)	Recovery (%)
		0.16	0.356 ± 0.011	97.61%
Posterior	0.20	0.22	0.432 ± 0.019	105.37%
silk gland		0.26	0.491 ± 0.022	111.92%

## Data Availability

The data presented in this study are available upon request from the corresponding author.

## Sample Availability

Samples of the compounds are available from the authors.
